# Exploring moral competence regression: a narrative approach in medical ethics education for medical students

**DOI:** 10.1186/s12910-024-01073-5

**Published:** 2024-06-21

**Authors:** Martin Zielina, Jaromír Škoda, Kateřina Ivanová, Daniel Dostál, Lubica Juríčková, David Anthony Procházka, Barbora Straka, Adam Doležal

**Affiliations:** 1 Department of Medical Ethics and Humanities, Second Faculty of Medicine, Charles University, V Úvalu 84, Prague, 15006 Czech Republic; 2 Department of Public Health, Faculty of Medicine and Dentistry, Palacký University Olomouc, Olomouc, Czech Republic; 3 Department of Psychology, Faculty of Arts, Palacký University Olomouc, Olomouc, Czech Republic; 4 First Faculty of Medicine, Institut for Medical Humanities, Charles University, Prague, Czech Republic; 5 Department of Paediatric Neurology, Second Faculty of Medicine, Charles University and Motol University Hospital, Prague, Czech Republic

**Keywords:** Moral competence, Medical students, Narrative approach, Konstanz Method of Dilemma Discussion, Problem-based learnig, Case-based learning

## Abstract

**Background:**

Studies from different countries report a stagnation or regression of moral competence in medical students between the first and the last year of their studies, and the value of various educational interventions remains uncertain.

**Methods:**

We used Moral Competence Test (MCT) to measure C-scores of moral competence to determine the change in the MCT C-scores between the first- and the fifth-year medical students from two medical schools in the Czech Republic in the academic year 2022/2023 and to analyze factors associated with the C-scores (observational study). In addition, for the first-year students, we compared the results of the MCT before and after an intervention in medical ethics curriculum (interventional study). We used a cross-sectional and descriptive design for the observational study. Students completed the MCT, consisting of two moral dilemmas (Worker´s Dilemma and Doctor´s Dilemma), the results measured by the C-score, which represents moral competence.

**Results:**

In total, 685 students participated in the observational study. Objective 1: based on the analysis of the C-score, we observed a decrease in moral competence between the first and the fifth-year medical students (*p* < .001). Objective 2: we did not observe a statistically significant effect of gender (*p* = .278), or self-rated religiosity (*p* = .163). Objective 3: in the interventional study, 440 students participated in the pretest and 422 students participated in the posttest. The test of statistical significance found no improvement in students’ moral competence after the intervention (*p* = .253).

**Conclusion:**

Medical students show a regression in moral competence during medical education; it was lower in medical students in their fifth year, compared to the first-year medical students without the effect of gender, or self-rated religiosity. Although educational intervention consisting of multiple tools of medical ethics teaching (PBL, CBL, KMDD and StorED) did not lead to increase in moral competence, the longitudinal effect of such intervention remains to be seen.

**Supplementary Information:**

The online version contains supplementary material available at 10.1186/s12910-024-01073-5.

## Background

Since the late 1970s, with the advent of modern technology and patients demanding their right to make treatment decisions based on their own values, medicine has been laden with emerging moral dilemmas [1]. In particular, ethical issues related to communication became painfully pronounced during the COVID-19 pandemic (e.g. ethical issues in informing the public about the risk of the pandemic, increasing inequities, stigmatization, ageism) [2], but many others have always been present in everyday clinical decisions. These include topics such as end-of-life care, care limitations, pre- and perinatal care, increasing healthcare costs, indications for genetic testing and others [3].

The skill to effectively handle ethically dilemmatic situations and to resolve them in accordance with internally held and socially accepted moral norms, has been described by many authors as moral competence [4]. Georg Lind defines moral competence as an individual´s ability to solve problems and conflicts solely through deliberation and discussion, without using violence and deceit, or submitting to an authority [5]. Lind was influenced by the cognitive-developmental approach stemming from Piaget’s [6] and Kohlberg’s [7] theories. Based on them, he formulated his own Dual Aspect Model [5], consisting of the affective and cognitive aspects, the latter of which can be measured by a C-score representing moral competence. To this end, Lind constructed the Moral Judgment Test (MJT) and its updated version, the Moral Competence Test (MCT). The test has been translated into multiple languages [8] and represents one of the most widely used tools to measure moral competence [4]. There have been discussions about whether gender or religiosity [9–11] influence the degree of moral competence. The influence of gender on moral judgment is an ambiguous and controversial topic [11–13]. In addition, according to Nowak et al. [11], the Doctor’s Dilemma inherent in MCT can be challenging in two contexts: religious and medical. We also investigated whether the speed of test completion or the perceived difficulty of the test has an effect. This is especially relevant in the context of an intuitionist approach to morality that sees our actions as merely “very quick and easy” post-hoc rationalizations of our emotions [14].

Competency-based frameworks are being promoted in contemporary medical education, whereby medical education is no longer understood as a process representing knowledge acquisition and time-limited clinical rotations, but as a process in which competencies can be understood as the adequate performance of a set of professional roles [15]. According to the Canadian CanMEDS model, such roles include: medical expert, communicator, collaborator, manager, health advocate, scholar, and professional [16]. And it is for the professionalism of physicians that moral competence plays a key role. According to Verkerk and others [17], reflective professionals possess the conscious and moral skills needed to be able to justify their actions.

Education influences moral competence more than other variables [18]. Despite this finding, Lind [19], in his longitudinal study reported a stagnation of moral judgement competence in medical students in Germany between the first and the final study year. This negative dynamic contrasted to the one observed in students of other fields, e.g. psychology, and this observation was confirmed by multiple authors [9, 11, 20–23]. In addition, other studies provided evidence that classical ethics education for medical students does not increase moral competence [24–26]. However, no study to date has compared the difference in moral competence between the first- and the fifth-year medical students, neither has provided solid evidence for the role of educational intervention consisting of a combination of multiple teaching approaches on moral competence of the first-year medical students.

According to Musick [27] medical ethics education has two main categories – 1) enhancing physicians´ conscience, good character and integrity and 2) the practice of clinical medicine including patient cases. Moral competence is rather a part of the first category. Moreover, narrative ethics is appropriate for the second category, since it does not consider clinical decisions as something isolated from everything else that happens to patients, but these decisions are part of an ongoing narrative [28]. There is no single ‘best’ method by which ethics should be taught [29]. Problem-based learning [30], case-based learning [31], storytelling [32] or the Konstanz Method of Dilemma Discussion [26] are all used in medical education. Although case-based methods are popular in medicine, the results are inconclusive in terms of the impact on learning compared to other types of activities [31].

In the presented study, we aimed to analyze the difference in moral competence of medical students at the beginning (1st year) and close to the end (5th year) of their studies. Next, we examined the extent to which the level of moral competence is related to other variables such as gender, age, self-rated religiosity, test completion duration and self-rated test difficulty. Finally, we analyzed whether an intervention in the form of medical ethics course with a narrative approach in the first year of medical studies affects moral competence.

## Methods

### Study design

The study was conceptualized with two aims in mind. First, we performed an observational cross-sectional study in which we compared the C-scores obtained in Lind’s Moral Competence Test (MCT) at the beginning of the first and fifth years of medical studies, and analyzed variables potentially affecting these scores. Second, we performed an interventional study in which we analyzed the effect of changes in the first-year medical ethics curriculum on C-scores in MCT before and after completion of the medical ethics course. In this study, first-year students were required to complete MCT twice: once prior to and once following the medical ethics course.

### Study population

We included the first and the fifth-year medical students of the academic year 2022/2023 from the Second Faculty of Medicine of Charles University (SFM CU) in Prague, Czech Republic and the Faculty of Medicine and Dentistry of Palacký University Olomouc (FMD PU) in Olomouc, Czech Republic. For the interventional study, the first-year medical students were tested at the beginning of the semester (pretest) and after attending the obligatory medical ethics course comprising 10 class hours (i.e. 450 min). Participation in the research was anonymous, voluntary and without remuneration.

The research project was approved by the Ethics Committee for Multicenter Clinical Trials of the University Hospital in Motol and the Second Faculty of Medicine of Charles University in Prague (EC − 380/22) and the Ethics Committee of the Faculty of Medicine and Dentistry of Palacký University Olomouc in Olomouc and University Hospital Olomouc (No. 136/22).

### Measures

Participants completed a short questionnaire on demographic and study-related variables (see details in the *Statistical Analysis* section), in addition to Lind’s Moral Competence Test (MCT).

The standard version of the MCT contains two scenarios: the Worker’s Dilemma and the Doctor’s Dilemma, in which the protanogists make decisions. Participants evaluate quality of arguments offered in these scenarios. Each dilemma is presented as a short story that allows for the evaluation of a total of 12 arguments, with 6 arguments supporting and 6 arguments opposing the main character’s decision. Each argument is developer to represent the six different types of moral orientation (see Supplement). The MCT score, also known as the Competence score or C-score, quantifies the proportion of variability in individual responses that can be explained by an individual’s ability to assess arguments in terms of their moral quality. C-scores range from 0 to 100 [5]. After completing the test, respondents were asked to rate how difficult they found it to evaluate moral dilemmas on a scale from 1 to 8 and to report the time it took them to complete the test.

### Intervention

Between the pre-test and post-test, an ethics course of 10 class periods was conducted in which ethically-dilemmatic case reports were discussed through various teaching methods: problem-based learning (PBL) and case-based learning (CBL) [33], learning inspired by Konstanz Method of Dilemma Discussion (KMDD) [5] and storytelling (StorEd) [34] (for detailed information on each of these teaching methods see Supplement). PBL is based on an open questioning approach. The CBL method is a guided inquiry method and provides more space for small group sessions during the learning process [35, 36]. Both CBL and PBL utilize general instructions while simultaneusly using a story about individuals [37]. When applying PBL in a medical ethics course, we followed these consecutive seven steps: clarify terms and concepts which are not clear; define the problem(s); analyze the problem (brainstorming); list possible explanations; formulate learning objectives and set priorities; look for additional information outside the group; report back, synthesise and test information [38]. In addition, in PBL, students are suddenly confronted with a clinical moral dilemma during class and must quickly find a solution to the presented problem. In CBL on the other hand, medical students know the dilemma in advance so that they can prepare for their ethical argumentation and defense of their ethical position [39].

In contrast to PBL and CBL, the presentation of a short story with moral dilemma through storytelling offers an artistic processing of the story. Storytelling is an art form in which trained drama artists narrate a story (an ethically dilemmatic medical case report in this study) in front of an audience or on a video recording. Except for the artists’ dramatic skills, storytelling makes use of no additional equipment (e.g. scene setting, costumes, music). The story is therefore presented in an intensive distilled form that should increase the suggestive and emotional effect on the participants. Storytelling enables the students to focus on the emotional experience of all involved participants of the story equally (e.g. the patient, the patient’s relatives, members of the medical team) and to appreciate their distinct perspectives. KMDD is an educational and psycho-didactic method designed to develop moral competence developing the following key abilities: individual moral autonomy, the ability to stand up for one’s own point of view and the capacity to listen to others when issues are at stake which are important to oneself or the other person. This also means the ability to look for and to maintain communication with others when strong moral feelings are involved on both sides. KMDD helps develop students’ abilities based on the discussion of semi-real moral dilemmas [5]. Unlike the methods mentioned above, these stories do not have to be dilemmas from a medical environment. For the purpose of PBL, CBL and StorED we obtained anonymized case reports from medical professionals in their postgraduate training as a part of the grant project TACR ETA no. TL05000114.

Before instructing at the medical schools, a comprehensive plan was created and reviewed, dividing study groups into those with planned interventions and those without. Furthermore, while both faculties used a common method called StorED, FMD PU employed PBL and CBL, and SFM CU incorporated KMDD into their interventions. The working teams at both medical schools used the same ethically controversial case studies and relied on the same interdisciplinary expert opinions, which consisted of the following parts: ethical issues, medical assessments, ethical assessments, and legal assessments. Both working teams also used the bioethical methods of principlism [40] and the casuistry method [41].

### Statistical analysis

The analyses were performed in R (4.3.0) using the lme4 [42] and emmeans [43] libraries. The hypothesis of a difference in C-score levels was tested using a linear regression model. The model included these factors: year of studies (first, last), medical school (SFM CU, FMD PU), gender (male, female), and three continuous covariates: self-rated religiosity (4-point ordinal scale), test completion duration (in minutes) and self-rated test difficulty (8-point ordinal scale). MCT C-score is computed as a sums of squares ratio and is therefore naturally squared, which affects its distribution. To achieve maximum statistical power and to meet the assumptions of parametric statistics, we used square root of the C-score as the dependent variable. However, we present the results transformed back in the original squared form.

Individual dilemma C-scores were calculated analogously to the total C-score, but only half of the test items were used each time. We therefore distinguish between C_W_ (Worker’s Dilemma) and C_D_ (Doctor’s Dilemma). As in the previous case, we performed the calculations on the square roots of the individual dilemma C scores. Two individual dilemma C scores (C_W_ and C_D_) were obtained from each participant. Consequently, the analysis involves repeated measures, so we used a mixed-effects approach with a random factor proband (657 levels).

In addition to statistical significance, we also present standardized regression coefficients β as indicators of the effect size. Effect sizes |β| between 0.10 and 0.29 indicates small effects, |β| between 0.30 and 0.49 indicates medium effects, and |β| of 0.50 or greater indicates large effects [44]. In the interventional part of the study, the effectiveness of the intervention was tested using a linear regression model. In addition to the measurement regressor (pretest, posttest), the model included the same set of factors (regressors) as in the observational study. For the reasons described above, the square root of the MCT C-score was used as the dependent variable. Due to the strictly anonymous nature of participation in the study, the recording sheets were not labeled with names or any identifying codes, and thus it was not possible to treat the data as paired measurements. We therefore treated the measurements as independent observations, although this procedure may have reduced the statistical power of the tests used.

## Results

### Characteristics of the study population

The observational part of the study involved 685 first- and fifth-year medical students at the SFM CU and the FMD PU. Nineteen participants were excluded due to incomplete questionnaires, additional 4 because their age significantly exceeded (above one standard deviation) the average age of the other participants in their respective groups (i.e. 34, 40, 41, 44 years), and another 5 who identified their gender as ‘other’ to increase homogeneity of the group. Descriptive characteristics of the study population are summarized in Table 1.

**Table 1:** Descriptive characteristics of the observational study research sample

Group		Frequency			Age	
		Women	Men		Range	M (SD)
*SFM CU*						
First year		115	75		18–27	19.83 (1.22)
Last year		56	50		23–30	23.97 (1.14)
*FMD PU*						
First year		148	91		18–28	20.04 (1.29)
Last year		83	39		23–29	24.12 (1.22)
*Total*		402	255		18–30	21.37 (2.31)

The interventional part of the study involved the first-year medical students at the SFM CU and the FMD PU. 440 students participated in the pretest and 422 students in the posttest. Due to incomplete questionnaires or age over 30, 6 observations in the pretest and 16 in the posttest were discarded. A further 8 participants (5 in pretest, 3 in posttest) reported the gender ‘other’, which, as in the previous study, is too small group to be included in the analysis. The data obtained partially overlap with the data used in the observational study (see Table 2 for details).

**Table 2:** Descriptive characteristics of the interventional study research sample

Group		Frequency			Age	
		Women	Men		Range	M (SD)
*SFM CU*						
Pretest		115	75		18–27	19.83 (1.22)
Posttest		114	66		18–27	19.91 (1.17)
*FMD PU*						
Pretest		148	91		18–28	20.04 (1.29)
Posttest		141	82		18–29	20.22 (1.31)
*Total*						
Pretest		263	166		18–28	19.95 (1.26)
Posttest		255	148		18–29	20.08 (1.26)

Note. The observations in the rows labeled pretest are equivalent to the subsample of first-year students from the observational study

### Objective 1: Moral competence is lower in the fifth-year medical students than in the first-year students

**Fig. 1:**
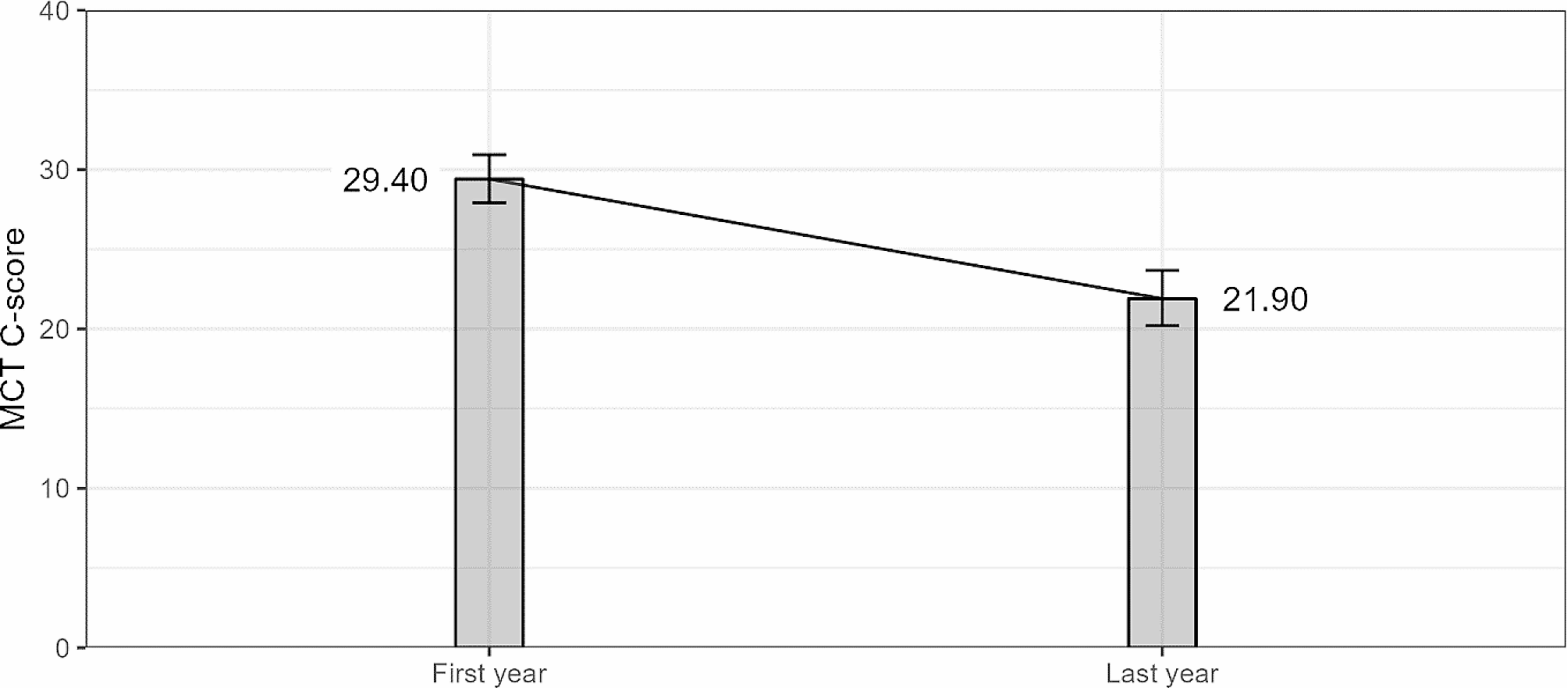
C-scores in students of the first and last year of medical studies. Note. Data presented are estimated marginal means after having controlled for covariates. For continuous regressors, the average of the covariates was assumed, for categorical factors it is the average across all their levels. Error bars indicate 95% point-wise confidence intervals

Medical students achieved a mean C-score of 26.56 (SD = 15.58), described with five-number summary: 1-15-24-36-81. The C-score showed positive skewness (0.75), however, the square root of the variable was almost perfectly symmetric (skewness = 0.004). The statistical model confirmed the hypothesis of a regression in moral competence during medical studies, t(650) = 6.339, β = − 0.238, *p* < .001. Students on average scored 29.40 at the beginning of their studies and 21.90 at the end (see Fig. 1).

### Objective 2: Moral competence is not influenced by gender or self-rated religiosity

We did not observe a statistically significant effect of neither gender, t(650) = 1.087, β = 0.041, *p* = .278, nor self-rated religiosity, t(650) = 1.396, β = 0.053, *p* = .163. A significant difference existed between schools with students at the SFM CU scoring 3.61 points higher on average, t(650) = 3.165, β = 0.120, *p* = .001. However, that the inclusion of the year of studies × school interaction does not lead to a more precise model, so there is no significant difference in the magnitude of the regressions in moral competence between schools, F(1, 649) = 1.402, *p* = .237. The regressors “test completion duration”:, t(650) = 2.132, β = 0.082, *p* = .033, and “self-rated test difficulty”, t(650) = 3.360, β = 0.130, *p* < .001, were statistically significant, although the effect sizes ranged from negligible to small. Students who spent more time completing the test and rated it as more difficult achieved slightly higher C-scores.

**Fig. 2:**
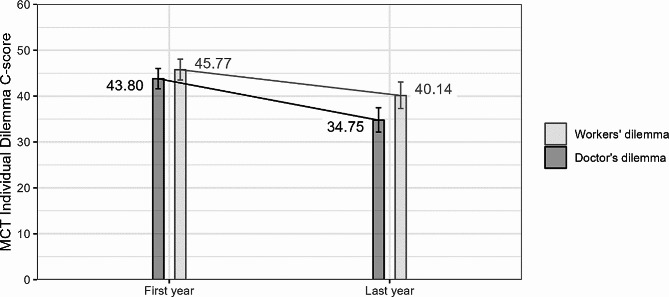
Individual dilemma C-scores in students of the first and last year of medical studies. Note. Data presented are estimated marginal means after having controlled for covariates. Error bars indicate 95% point-wise confidence intervals. Note that the individual dilemma C-scores are on average higher than the overall C-score. This is not an error, but a result of the C-score being generated not as a linear combination of the responses of the respondent, but as a sum of squares ratio

Analysis of individual dilemma C-scores confirmed the regression in moral competence in both the Worker’s Dilemma, t(1317) = 2.993, β = 0.114, *p* = .003, and the Doctor’s Dilemma, t(1317) = 5.057, β = 0.194, *p* < .001. Although the regression was higher for C_D_ than for C_W_, there is no statistically significant difference between the effect sizes, F(1, 656.5) = 2.298, *p* = .130. This suggests that the regression in moral competence during the medical studies is not exclusive to dilemmas from the medical environment (see Fig. 2).

### Objective 3: Intervention in medical ethics curriculum does not affect moral competence in the first-year medical students

**Fig. 3:**
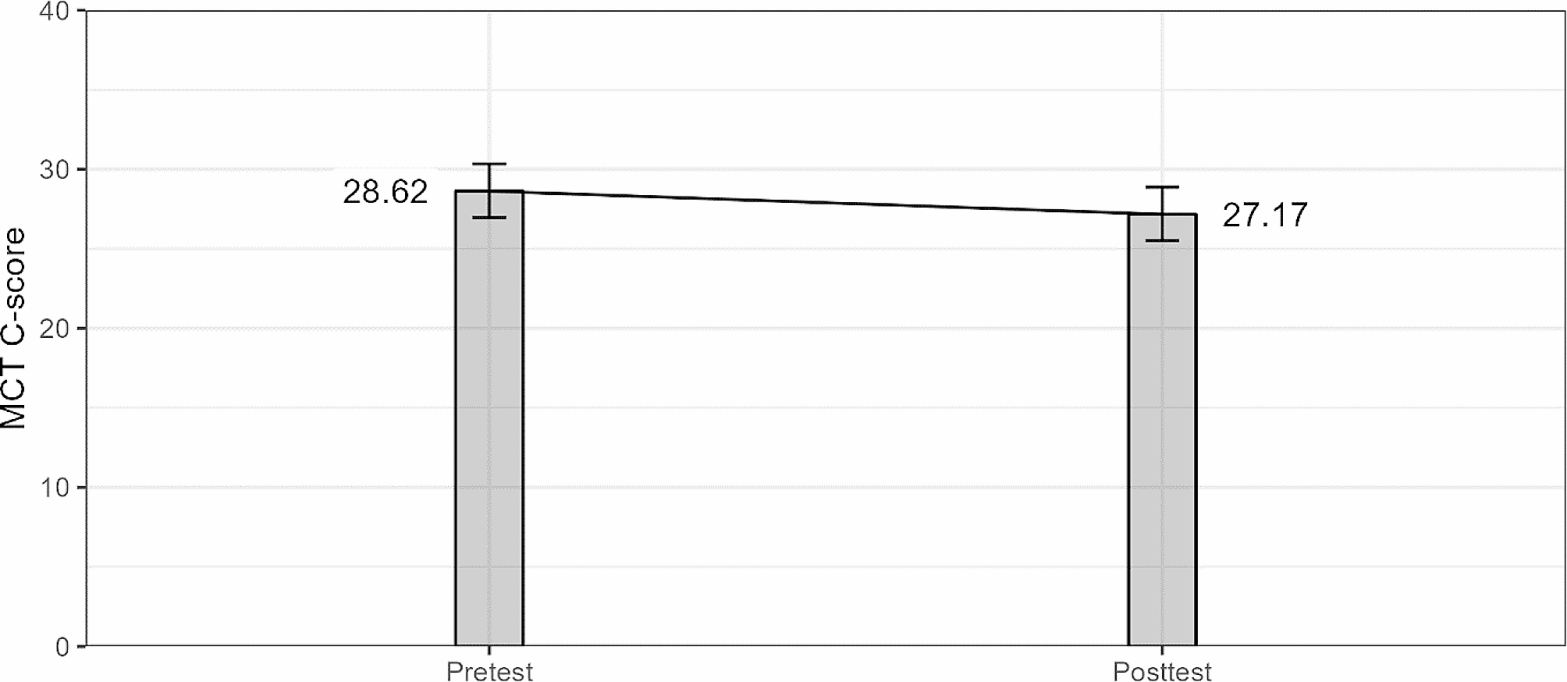
C-scores in students of the first year of medical studies before and after the intervention. Note. Data presented are estimated marginal means after having controlled for covariates. Error bars indicate 95% point-wise confidence intervals

MCT C-scores have similar distribution as observed in the observational study. Described with 5 numbers 0-15-26-39-81, with mean 27.93 and standard deviation 16.42. Again, a positive skew (0.59) was present. The test of statistical significance did not detect an improvement in the moral competence of the students after the intervention, t(825) = 1.145, β = 0.044, *p* = .253; moreover, the mean value decreased slightly (from 28.62 to 27.17 points), although not significantly (see Fig. 3).

The addition of the measurement × school interaction allowed us to examine whether there are differences in the change in moral competence between medical schools. The results indicate a nearly identical regression in both subsets, F(1, 824) = 0.023, *p* = .879. Students at the SFM CU deteriorated by 1.31 points, and students at FMD PU deteriorated by 1.57 points.

## Discussion

In the presented study, we found that moral competence, as measured by C-scores of the MCT test, was significantly lower in the fifth-year medical students compared to those of their counterparts in the first year. None of the analysed factors (regressors) significantly influenced the C-scores or their effect sizes ranged from small to negligible. We also found that the tested interevention in the medical ethics curriculum in the first-year medical ethics course with a narrative approach did not affect the C-scores in the first-year medical students.

### Moral competence decreases in the course of medical studies

Our findings confirm those of other researchers who have shown a regression of moral competence in medical students between their first and final years [9, 11, 20–23]. Multiple explanations of this phenomenon have been proposed. First, a number of studies highlight the role of idealism and humanistic orientation at the beginning of medical studies, replaced by pragmatism and loss of ideals [9], exhaustion, stress, the presence of depressive episodes and crises [11], a decrease in empathy and an increase in cynicism [45] in the final years of medical studies; in addition, hidden curriculum may also play a role [21]. Hegazi and Wilson [9], hypothesise that the purported regression in moral competence relates only to dilemmas from the medical setting that health professionals may judge differently from the general population, a phenomenon they describe as moral segmentation. Moral segmentation occurs when there is a decrease in C-scores of 8 or more points calculated for each dilemma separately. Thus, moral segmentation could contribute to an overall regression in moral competence. We observed a decrease in both the overall C-Score of the MCT (see the Measures section) and the subscores derived separately from responses related to the Worker´s and Doctor´s Dilemmas. While our results correspond to the trend observed by Hegazi and Wilson, the differences are not statistically significant and thus the regression in C-score for senior medical students cannot be attributed to the phenomenon of moral segmentation alone.

Religion may also contribute to moral segmentation [21], which may support the claim that moral competence is not solely an individual category but is also influenced by socio-cultural factors. Biggs and Colesante [10] have shown that men had a lower C-score than women, and for men, the relationship of the C-score to religiosity was positive, whereas for women, it was negative. However, our results showed no statistically significant difference with respect to gender or self-reported religiosity.

Finally, a ceiling effect was proposed as a potential explanation for the lack of increase in moral competence in medical students. The ceiling effect would suggest that moral competence is already so high among medical students that it would be impossible to increase it further, a hypothesis later rejected by Lind himself [19]. In contrast, our results, in accordance with the above-mentioned studies, show that moral competence decreases during medical studies.

### Intervention in medical ethics curriculum did not affect C-scores in the first-year medical students

Given the evidence for the decrease in moral competence in medical students, multiple authors have proposed interventions aimed at increasing moral competence among healthcare students. Nowak and others [11] report a 6.4-point improvement in C-scores for 115 Polish healthcare students using problem-based learning. On the other hand, according to Gomes and Rego [46], problem-based learning is not a suitable method for increasing moral competence. This discrepancy seems to reflect a difference in the context of the learning environment, which in the case of Nowak and others [11] included a simulated patient, role-taking, responsibility-taking and guided reflection. According to Zhao and others [47], it is appropriate to combine problem-based learning emphasizing the student’s initiative and case-based learning supporting this initiative with teacher-created discussion questions. The combination of problem-based learning and case-based learning can be successfully used in medical education, as confirmed by Zhao and others [47]. The results of our study showed no statistically significant effect of using the combination of problem-based learning and case-based learning on moral competence. The clear discrepancy between our results and those of the above-mentioned authors might stem from multiple factors. First, medical students in the Czech Republic tend not to engage strongly in in-class discussions and may need more encouragement to participate, express their opinions and to discuss the proposed ethical dilemmas. Second, the study of medicine is still anchored in a strongly positivistic perspective, a possible reflection of a hidden curriculum [48], that does not allow much space for multiple non-exclusionary approaches towards a proposed dilemma and argues for a single definite solution, a factor associated with lower C-scores in MCT. Third, medical students tend to view medical ethics as a “humanities” course and as such consider it less valuable and useful for their future medical practice. Finally, an increase in moral competence was associated with an appropriate learning environment (i.e., role-taking and guided reflection) in the above-mentioned studies, but the analysis of learning environment was not the focus of our study.

We were able to test the effect of the intervention on moral competence in the first year, in contrast to the fifth, because medical ethics is taught only in the first, usually two, years of medical curriculum. Therefore, the presented data on a sample of Czech medical students can contribute to the discussion on the appropriateness of including ethics courses in the later years of medical studies.

### The role of the Konstanz Method of Dilemma Discussion in fostering moral competence

Georg Lind [5] developed the Konstanz Method of Dilemma Discussion (KMDD) for increasing moral competence. Lerkiatbundit and others [49] reported an increase in moral competence using this method over a control group in Thai health students and Serodio and others [26] in Brazilian medical students. To increase moral competence, it is important to ensure an appropriate learning environment where role-taking and guided reflection play an important role [22], especially when students take responsibility in both dimensions [5]. It remains to be analyzed whether a 450-minute course in ethics is too short to increase moral competence, or whether and how the methods and/or content of the course precluded the increase. In addition, the type of learning environment (i.e. role-taking and guided reflection) seems to play a very important role, which requires a major effort and experience of the teachers.

### Practical implications

Fostering moral competence is not the sole purpose of medical ethics education. In their immediate feedback at the end of the sessions on ethically-dilemmatic cases, medical students’ assessment was overwhelmingly positive and they found the lessons enjoyable. In discussing moral dilemmas, students were confronted with the fact that there are different or even opposing views to the ones they hold. During these discussions, students very often expressed strong emotions or helplessness when confronted with an opposing viewpoint. At the same time, students were encouraged to engage in a democratic discussion in which opposing views can be voiced without escalating conflict [5]. In addition, the opportunity to express their views encouraged students not to agree at all costs, but to seek a solution that best reflects the views of all stakeholders while complying with the current legislation. We believe that ethically-dilemmatic cases that are memorable, multidimensional and emotionally intense have the power to influence students’ professional and moral development [50].

## Limitations

Our study has certain limitations. First, the comparison groups of first and fifth year students at the two schools may differ significantly from each other given that we do not have information on the level of C-scores for fifth year students when they were at the start of their studies. Nevertheless, it appears that, as a rule, there is a regression of C-scores over time for medical students as they progress through their studies [9]. Second, medical ethics courses took place at the two respective medical schools, and therefore, certain differences in approach could not have been avoided. Each of the educators (MZ, KI, AD, JS, LJ) might have preferred either or none of the narrative educational approaches (e.g. KMDD, PBL, CBL, storytelling). In order to secure the alignment and comparability of the intervention in medical ethics curricula between the two participating centers, over the study period, we conducted multiple workshops during which the respective teams discussed the details of the reported teaching methods (detailed descriptions of the methods below). The teams utilised the same storytelling video recordings and selected members from both teams participated in a dedicated course on KMDD by the co-authors of the methods. Both PBL and CBL rely on the group dynamics and the personality of the instructor, therefore, the role of either institution per se was limited.

However, given the large sample size and the participation of multiple educators, we believe that these differences did not significantly affect the outcomes. In addition, the medical school where teaching took place was included in our statistical analyses as one of the factors (regressors), and it did not reach statistical significance. Third, in the Czech system of medical education, students enter clinical practice in the fourth year of medical studies, with the first three years focused on theoretical subjects, e.g. anatomy, physiology, biochemistry, pathology and others. Therefore, some case reports might have been difficult to relate to in the first year. Finally, even though the MCT represents a well-recognized and widely used tool for measuring moral competence, participant-related factors (e.g. personal motivation, level of tiredness, time of the day) might have influenced the scores. Finally, even if there was no increase in moral competence in the first year, it remains to be found whether and how the presented intervention in medical ethics curriculum will affect moral competence in further years of medical studies, and therefore a longitudinal follow-up study is warranted.

## Conclusion

Moral competence is lower in medical students close to the end of their studies, compared to the first-year medical students without the effect of gender, or self-rated religiosity. Although educational intervention consisting of multiple tools of medical ethics teaching (PBL, CBL, KMDD and StorEd) did not lead to increase in moral competence in the first-year medical students post-intervention, the students valued the intervention. The longitudinal effect of such intervention remains to be seen.

### Electronic supplementary material

Below is the link to the electronic supplementary material.

Supplementary Material 1

## Data Availability

The datasets analyzed during the current study are available from the corresponding author on reasonable request.
