# Supplementary material for: Exploring moral competence regression: a narrative approach in medical ethics education for medical students
Source: BMC Med Ethics. 2024 Jun 21;25:73. doi: 10.1186/s12910-024-01073-5 (PMC11191321; doi:10.1186/s12910-024-01073-5)
Supplement: Supplementary file 1 — Supplementary Material 1 [file 12910_2024_1073_MOESM1_ESM.doc]

**Supplementary data**

Below, we present detailed characteristic of the Moral Competence Test (Chapter 1) the educational methods utilized in the intervention, highlighting the primary methods applied at FMD PU, predominantly PBL and CBL (Chapter 2), at SFM CU KMDD (Chapter 3), and at both medical schools StorEd (Chapter 4). These descriptions constitute a detailed methodological framework for implementing ethical education in medical faculties, accessible on the website: www.etikamedika.cz.

# 1. Detailed characteristic of the Moral Competence Test (MCT)

The MCT is a behavioral experiment, measurment tool, which allows to measure internal, structural properties of person´s behavior in an objective way. It is based on Lind´s Dual-aspect model of moral behavior theory which consists from moral competence (cognitive aspect) and moral orientation (affective aspect). Moral orientation means internal force which which directs and energizes behavior, like attitudes, values, motives, postures, etc. [1]. MCT scores, in agreement with this, are classified in two aspects: cognitive and affective aspect.

Moral competence is expressed by the C-score. The C-score (competence score) obtained from the MCT scoring in its cognitive aspect reflects the participant’s ability to judge arguments according to their moral quality [1]. A high moral competence shows itself when arguments are valued after a moral quality independent from the type of dilemma and not according to the own opinion. A low moral competence is given if the tested person rather values the arguments according to the own opinion. He or she is not capable of taking a distance to the own opinion and to value arguments for their moral quality. The C-Score ranges between 0 and 100. A C-Score of 100 means that 100% of the variance can be explained by orientation to moral type, which means that the person orients completely to the moral quality of the arguments.

Another feature of the MCT is the assessment of the affective aspect which is reflected through a person’s attitudes towards each of the six types of moral reasoning [2]. The preference for the six types is scored in order to test the criteria of hierarchical order of stages and affective-cognitive parallelism [3]. The affective aspect is, therefore, not reflected by the C-score. The cognitive aspect as measured by the MCT is independent of a person’s moral attitudes.

This test assesses an individual's moral competence, although it is mainly designed more for group assessment [1]. Through this instrument, one can assess not only the growth of moral competence, but also any regression. The MCT is designed as three-factor (6 x 2 x 2) N = 1 experimental design. Its factors are: Moral Quality of Arguments (six types of moral orientation), Opinion-Agreement with Arguments (two types of pro-contra factor, i.e., orientation to agree/disagree with the given arguments according to the proband) and Dilemma-Context of Arguments (two types of dilemma factor, i.e., orientation to the content of the stories).

Each dilemma in story form thus allows for the evaluation of a total of 12 arguments, 6 of which are in support of and 6 against the story dilemma. Each of these arguments consists of all three of the above factors. Lind's test is based on two types of moral dilemma, the Worker's Dilemma and the Doctor's Dilemma. The possibility of replacing one dilemma was also discussed in the literature. However, for such a dilemma to be adequate, it must be a difficult moral task. For adequate moral competence test it is necessary for the participants to engage their ability to think and discuss opposing views on moral issues [1].

The Moral Competence Test is protected by copyright, however, for research purposes it is possible to contact the copyright holder via the www.moralcompetence.net website as well as to find further information about the test. With the consent of the author of the test, George Lind, we have provided a Czech translation of the revised version of the test.

# 2. Problem-Based Learning (PBL), Case-Based Learning (CBL)

## 2.1 PBL and CBL - Common Context

Ethical dilemmas permeate all areas of medicine to varying degrees. Thus, it is essential in medical ethics education to clarify educational goals in cognitive, affective, and behavioral domains. To develop cognitive and behavioral attitudes effectively, it is beneficial to use a series of discussions based on quasi-real-life ethical dilemmas and to highlight fundamental principles related to medical practice. Didactic methods of Problem-Based Learning (PBL) and Case-Based Learning (CBL) use records of quasi-real patient problems as a context in which students acquire problem-solving skills and knowledge from basic and clinical sciences [4].

Both CBL and PBL represent broader education through individual stories [5-7]. These methods are desirable as the nature of ethical inquiry is highly compatible with the learning processes typical of PBL and CBL [8]. Many universities in Anglo-Saxon countries integrate the principles of PBL and CBL into their curricula. One of the first universities to start using the PBL method in the 1960s was McMaster University's medical faculty in Hamilton, Canada, implemented by Howard Barrows. PBL was assumed to provide students with a method to integrate knowledge across subject boundaries and develop problem-solving skills [9]. PBL/CBL methods also provide a broader context for understanding concepts of Corporate Social Responsibility (CSR) and solving ethically challenging cases and situations in healthcare. They deepen team discussions, help students apply basic scientific concepts to ethically controversial cases, enhance understanding of the complexities of patient care, and provide an authentic experience in gaining competencies for making ethically controversial decisions while fostering empathy.

## 2.2 Problem-Based Learning Method - PBL

The PBL method is an open-ended questioning approach focused on the problem-solving process. It is student-centered, focusing on the future physician and the ethical problem in medicine where ethical principles are integrated into the discussion [10]. This didactic method moves from defining the problem, proposing a solution, to practical patient care. It is an educational method utilizing quasi-real patient problems as a context for students to learn problem-solving skills and acquire knowledge about basic and clinical sciences [4].

PBL overlaps with Inquiry-Based Learning (IBL), where the teacher presents a problem, and students must develop inquiries to identify the problem. Students discuss and expand the range of topics related to the problem in class. The goal of the PBL educational process is to propose a method for problem-solving rather than detailed procedures. Therefore, focus is on the educational process, the questions asked, and student interactions.

PBL aims to identify the problem, determine what knowledge is needed for resolution, and then learn how to solve the problem in practice. Students encounter the problem during class, unprepared. PBL teaches students to actively find and explain the solution to a real situation or problem, not a specific case, although the problem may be shown on a specific case. Emphasis is on finding evidence for problem-solving through self-assessment or student collaboration, involving critical thinking. The instructor acts solely as a facilitator.

PBL is defined as an educational strategy where students confront contextual, structured problems and seek meaningful solutions. It is widely used in medical faculties to promote lifelong learning, open inquiry, teamwork, and critical thinking. Students are active participants in learning, not passive listeners. PBL encourages stimulating and productive discussions on shared understanding based on presented ethical dilemmas. Bligh [11] outlines seven steps in PBL education:

- Clarify terms and concepts which are not clear;
- Define the problem(s);
- Analyze the problem (brainstorming);
- Create a list of possible explanations;
- Formulate learning objectives and set priorities;
- Look for additional information outside the group;
- Report back, synthesize, and test information.

PBL advocates assert that it promotes lifelong learning, simulates clinical practice, enhances curiosity, and broadens understanding of medicine's complexity.

## 2.3 Case-Based Learning Method - CBL

CBL is a guided inquiry-based educational method. Students are presented with real or more often hypothetical cases simulating real scenarios and provided with a space to solve these cases in small groups. CBL aims for students to learn specific procedures for the given case or similar cases. The teaching design should enable students to learn as much as possible and in detail about the clinical case, diagnosis, treatment, or addressing ethical-legal questions.

The didactic method moves from specific, detailed cases to identifying a general problem that can be paradigmatic and applicable to similar cases [12]. The University of Pittsburgh defines CBL as general learning through individual stories or cases.

CBL requires partially informed or advanced students who need to prepare in advance for classes and discussions. Holden et al. [13] emphasize that students benefit from prior knowledge of the discussed topic. Students are expected to engage appropriately, prepare ahead, and ask relevant questions. Teachers should not only provide the case content but guide discussions or content delivery, ensuring specific educational goals are met.

Multiple cases can be presented, chosen to meet educational objectives. Learning outcomes are assessed to determine goal achievement. Students should have time to understand the case, propose solutions, defend their stance, or seek additional expert opinions or materials. They should identify the main problem cause, explore various solutions, and select the best one using knowledge and skills from education and research.

CBL is flexible, applicable in physical and virtual classrooms, legal arguments, business case studies, medical procedures, and more. It fosters understanding complex problems through previous solutions and optimized recommendations. It helps students develop critical thinking and problem-solving skills, as well as research and collaborative skills, supporting the creation of authentic solutions and recommendations for specific practice. CBL is commonly used in professional fields like medicine, law, business, engineering, and journalism.

# 3. KMDD® Teaching Method

KMDD® is an educational and psychodidactic tool focused on developing moral competence, which is crucial for fostering moral autonomy. The aim is to help students perceive moral issues and form their moral judgment within a broader societal context, promoting shared democratic values like justice, cooperation, freedom, and fundamental human rights [14].

Moral competence refers to the ability to solve problems and conflicts based on moral principles through reasoning and discussion, avoiding violent solutions and deceit. KMDD helps individuals understand their moral principles, articulate moral goals, and comprehend the moral objectives of others.

Supporting personal moral development is a significant and challenging task at all educational levels. KMDD involves discussing moral dilemmas in semi-realistic narrative stories, enhancing moral competence through several key abilities [14]:

- Recognizing and verbalizing one's moral feelings;
- Careful observation of case circumstances and specific facts;
- Differentiating between one's moral principles according to their importance and applicability;
- Identifying guiding principles to resolve conflicts;
- Presenting one's viewpoint even when opposed by others or friends;
- Providing opportunities for contemplation and discussion under emotional, social, or time pressure;
- Not just tolerating but valuing other perspectives, aiding in avoiding poor decisions.

# 4. Storytelling and Teaching Method StorED

## 4.1 Introduction – Converting Czech Case Studies into Stories (Storytelling)

The teaching method using storytelling and case studies from the Czech environment (StorED) is innovative and unique, incorporating artistic narrative elements to capture student attention. Ethical application in medical practice is complicated by factors like time pressure, healthcare worker fatigue, conflicting wishes, or emotional strain during difficult conversations. Storytelling aims to increase the emotional impact on participants, bringing problematic factors closer to students. Although video case studies or stories cannot convey these experiences fully, they have a stronger emotional impact than written case studies. Storytelling aligns with modern medical ethics trends, emphasizing the negative effects of advanced technologies and mechanized medicine, leading to depersonalization and dehumanization in medicine [15, 16].

Technical medical education often focuses solely on treating disease, neglecting the suffering person with their own story [17, 18]. Storytelling highlights the patient's story, emphasizing their values, preferences, and wishes as central to medical ethics. This artistic approach integrates bioethics, medical ethics, psychology, social sciences, and modern legal trends.

Story creation involves several steps. Initially, a dramaturge selects appropriate stories from ethically contentious case studies, assigning themes and keywords. Storytellers choose specific case studies to develop. The expert team provides additional information to create the narrative, addressing key questions such as the main message, ethical contention, and considerations for students.

Coordinators handle additional storyteller inquiries, providing expert responses and sending them to the dramaturge. Storytellers discuss the story's dramaturgical interpretation and overall solution, achieving a coherent narrative. The storytelling is written, reviewed, and visually recorded. After internal discussions and feedback, the final storytelling is produced, documenting the comprehensive narrative.

## 4.2 StorED as an Educational Tool with Czech Case Studies

StorED, prepared for the academic year 2022/2023, utilizes four specific Czech case studies crafted into storytelling recordings and is conducted at FMD PU (Olomouc) and at SFM CU (Prague). Teachers received interdisciplinary expert opinions comprising case description, ethical dilemmas, medical, ethical, and legal evaluations. Ethical assessments used principlism [19] and casuistic [20] methods. Expert-reviewed case studies are published on www.etikamedika.cz.

**Educational Method Description and Objectives**

StorED supports and enhances medical-ethical erudition, aimed at improving practical ethical decision-making skills. It helps students perceive ethical problems and find solutions based on current medical ethics, professional standards, and personal moral judgments. This erudition deepens understanding of bioethical principles like autonomy, beneficence, nonmaleficence, and justice, and applies casuistic methods in practice.

Medical-ethical erudition encompasses the capability to handle ethical dilemmas in medical practice, fostering preparedness to address ethically controversial situations.

Key competencies include:

- Perception of ethical problems and errors;
- Contextual understanding;
- Communication with patients and their families;
- Knowledge of ethical principles;
- Ethical problem categorization;
- Conflict resolution of principles;
- Application of ethical institutions to specific cases;
- Use of analogy with paradigmatic cases;
- Four-level casuistic method application;
- Seeing the patient as a person, not just a subject;
- Collaborative goal-setting with patients.

Medical-ethical erudition encompasses preparation to ethically navigate medical practice's complexities.

REFERENCES:

1. Lind G. How to teach moral competence. Berlin: Logos; 2019.

2. Kohlberg L. The Psychology of moral developmen. Essays on moral development. San Francisco, CA: Harper & Row; 1984.

3. Lind G. The Meaning and Measurement of Moral Judgment Competence Revisited: A Dual-Aspect Model. In: Fasko D, editor. Contemporary Philosophical and Psychological Perspectives on Moral Development and Education. Cresskill: Hampton Press; 2008. p. 185-220.

4. Eshach H, Bitterman H. From case-based reasoning to problem-based learning. Academic Medicine. 2003;78(5):491-6.

5. Srinivasan M, Wilkes M, Stevenson F, Nguyen T, Slavin S. Comparing problem-based learning with case-based learning: effects of a major curricular shift at two institutions. Academic Medicine. 2007;82(1):74-82.

6. Thiel CE, Connelly S, Harkrider L, Devenport LD, Bagdasarov Z, Johnson JF, Mumford MD. Case-based knowledge and ethics education: Improving learning and transfer through emotionally rich cases. Science and engineering ethics. 2013;19:265-86.

7. Tysinger JW, Klonis LK, Sadler JZ, Wagner JM. Teaching ethics using small-group, problem-based learning. Journal of Medical Ethics. 1997;23(5):315-8.

8. Heidari A, Adeli S-H, Taziki S-A, Akbari V, Ghadir M-R, Moosavi-Movahhed S-M et al. Teaching medical ethics: problem-based learning or small group discussion? Journal of medical ethics and history of medicine. 2013;6.

9. Barrows HS. Problem‐based learning in medicine and beyond: A brief overview. New directions for teaching and learning. 1996;1996(68):3-12.

10. Iramaneerat C. Moral education in medical schools. Journal of the Medical Association of Thailand= Chotmaihet Thangphaet. 2006;89(11):1987-93.

11. Bligh J. Problem-based learning in medicine: an introduction. Postgraduate medical journal. 1995;71(836):323-6.

12. Juríčková L, Ivanová K, Azeem K, Tučková D, editors. Teaching Communication with Disabled Patients Using Case-Based Learning–Experience from practice. 7th International Conference on Higher Education Advances (HEAd'21); 2021: Editorial Universitat Politècnica de València.

13. Holden BJ, Burnett AH, Vivekananda-Schmidt P. Case-based learning: integrating medical ethics and law into clinical practice. Education for Primary Care: an Official Publication of the Association of Course Organisers, National Association of GP Tutors, World Organisation of Family Doctors. 2014;25(5):283-7.

14. Lind G. How to teach morality: promoting deliberation and discussion, reducing violence and deceit. Logos Verlag Berlin GmbH; 2016.

15. Ramsey P, Jonsen AR, May WF. The patient as person: explorations in medical ethics. Yale University Press; 2002.

16. Rothman DJ. Strangers at the bedside: A history of how law and bioethics transformed medical decision making. Routledge; 2017.

17. Doležal T, & Doležal, A. . Informovaný souhlas ve zdravotnictví : právní a etické aspekty. Ústav státu a práva AV ČR, v.v.i.; 2023.

18. Gibson WM. Can personalized medicine survive? Canadian Family Physician. 1971;17(8):29.

19. Tom. L. Beauchamp JFC. Principles of biomedical ethics. New York, NY: Oxford University Press; 2019.

20. Jonsen AR, Toulmin S, Toulmin SE. The abuse of casuistry: A history of moral reasoning. Univ of California Press; 1988.
